# Left Ventricular Myocardial Septal Pacing in Close Proximity to LBB Does Not Prolong the Duration of the Left Ventricular Lateral Wall Depolarization Compared to LBB Pacing

**DOI:** 10.3389/fcvm.2021.787414

**Published:** 2021-12-07

**Authors:** Karol Curila, Pavel Jurak, Kevin Vernooy, Marek Jastrzebski, Petr Waldauf, Frits Prinzen, Josef Halamek, Marketa Susankova, Lucie Znojilova, Radovan Smisek, Jakub Karch, Filip Plesinger, Pawel Moskal, Luuk Heckman, Jan Mizner, Ivo Viscor, Vlastimil Vondra, Pavel Leinveber, Pavel Osmancik

**Affiliations:** ^1^Cardiocenter, Third Faculty of Medicine, Charles University, University Hospital Kralovske Vinohrady, Prague, Czechia; ^2^The Czech Academy of Sciences, Institute of Scientific Instruments, Brno, Czechia; ^3^Department of Cardiology, Cardiovascular Research Institute Maastricht (CARIM), Maastricht University Medical Center, Maastricht, Netherlands; ^4^First Department of Cardiology, Interventional Electrocardiology and Hypertension, Jagiellonian University, Medical College, Krakow, Poland; ^5^Department of Anesthesia and Intensive Care, Charles University, University Hospital Kralovske Vinohrady, Prague, Czechia; ^6^Department of Physiology, Cardiovascular Research Institute Maastricht, Maastricht University, Maastricht, Netherlands; ^7^Department of Biomedical Engineering, Faculty of Electrical Engineering and Communication, Brno University of Technology, Brno, Czechia; ^8^International Clinical Research Center, St. Anne's University Hospital, Brno, Czechia

**Keywords:** left bundle branch pacing, left septal myocardial pacing, UHF-ECG, dyssynchrony, depolarization duration

## Abstract

**Background:** Three different ventricular capture types are observed during left bundle branch pacing (LBBp). They are selective LBB pacing (sLBBp), non-selective LBB pacing (nsLBBp), and myocardial left septal pacing transiting from nsLBBp while decreasing the pacing output (LVSP). Study aimed to compare differences in ventricular depolarization between these captures using ultra-high-frequency electrocardiography (UHF-ECG).

**Methods:** Using decremental pacing voltage output, we identified and studied nsLBBp, sLBBp, and LVSP in patients with bradycardia. Timing of ventricular activations in precordial leads was displayed using UHF-ECGs, and electrical dyssynchrony (e-DYS) was calculated as the difference between the first and last activation. The durations of local depolarizations (Vd) were determined as the width of the UHF-QRS complex at 50% of its amplitude.

**Results:** In 57 consecutive patients, data were collected during nsLBBp (*n* = 57), LVSP (*n* = 34), and sLBBp (*n* = 23). Interventricular dyssynchrony (e-DYS) was significantly lower during LVSP −16 ms (−21; −11), than nsLBBp −24 ms (−28; −20) and sLBBp −31 ms (−36; −25). LVSP had the same V1d-V8d as nsLBBp and sLBBp except for V3d, which during LVSP was shorter than sLBBp; the mean difference −9 ms (−16; −1), *p* = 0.01. LVSP caused less interventricular dyssynchrony and the same or better local depolarization durations than nsLBBp and sLBBp irrespective of QRS morphology during spontaneous rhythm or paced QRS axis.

**Conclusions:** In patients with bradycardia, LVSP in close proximity to LBB resulted in better interventricular synchrony than nsLBBp and sLBBp and did not significantly prolong depolarization of the left ventricular lateral wall.

## Background

Left bundle branch (LBB) pacing is defined as the pacing of the trunk of the LBB or its proximal fascicles, usually with septal myocardial capture at low output (1). When pacing the LBB, three types of ventricular capture were identified during pacing maneuvers, i.e., decreasing the pacing output. The first is selective LBB capture (sLBBp), during which exclusively the LBB is captured. The second is non-selective LBB capture (nsLBBp), which is defined as concomitant LBB and adjacent left septal myocardial capture. The third is pure myocardial left septal capture (LVSP) which transits from nsLBBp during pacing maneuvers (1).

During nsLBBp, sLBBp, and LVSP, a QRS morphology with a right bundle branch block-like pattern is usually present in lead V1. However, this paced QRS pattern is also present in left septal positions that are shallower than positions where LBB capture could be observed during pacing maneuvers (2, 3). Our previous study used the ultra-high-frequency ECG (UHF-ECG) to show that myocardial capture of the left septum (in positions where nsLBBp was not obtainable with pacing outputs up to 5 V at 0.5 ms) produced less interventricular dyssynchrony but prolonged LV lateral wall depolarization durations compared to nsLBBp (4). The impact of pure myocardial left septal pacing using pacing positions, which are closer to the LBB, i.e., locations where left septal myocardial capture appears from nsLBBp while decreasing pacing outputs, is not known. Also, the impact of sLBBp on ventricular depolarization has not been described.

This study aimed to compare ventricular depolarization using UHF-ECG during LVSP, sLBBp, and nsLBBp in patients with bradycardia and an indication for pacing.

## Methods

### Study Design and Study Population

In this prospective study, consecutive patients with an indication for pacemaker implantation due to bradycardia were included. The project was approved by the Ethics Committee of the Faculty Hospital Kralovske Vinohrady, Prague, CZ; all subjects signed informed consent before enrollment.

### Pacemaker Implantation

**Figure 1:**
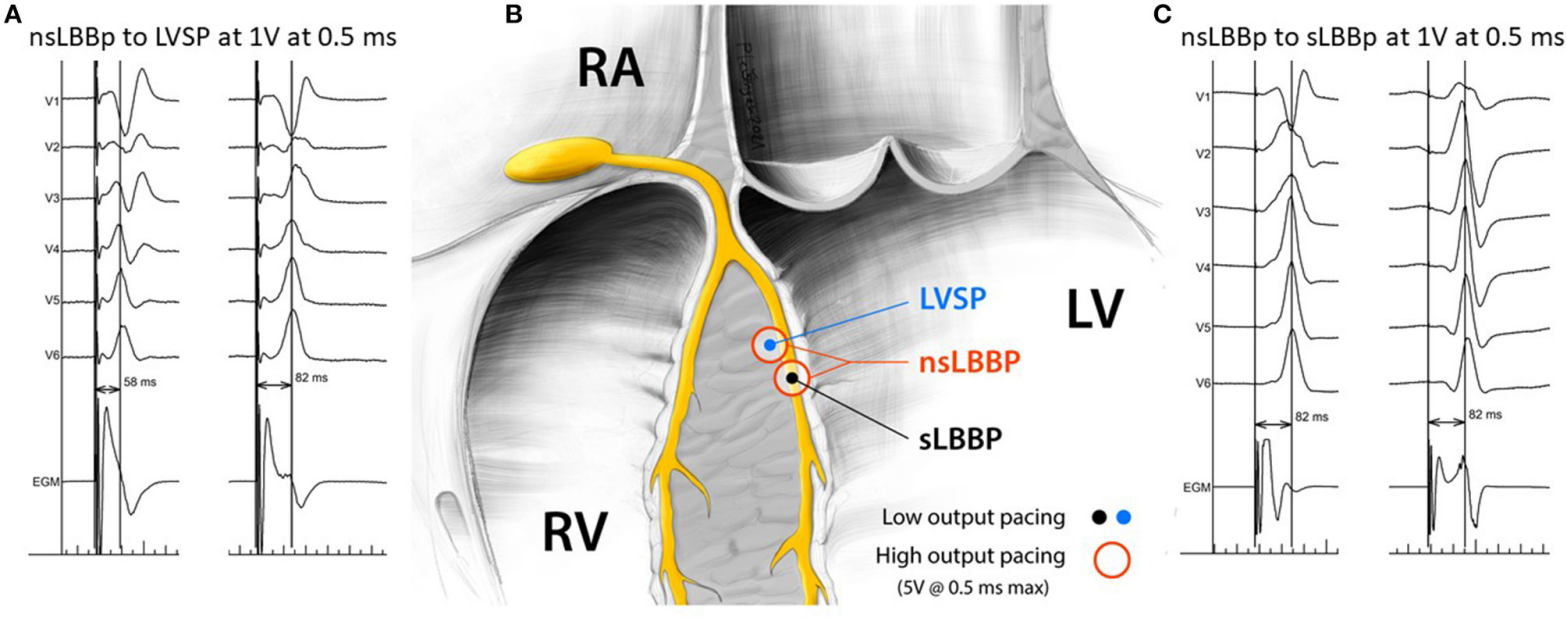
Visualization of nsLBB to LVSP and nsLBBp to sLBBp transitions during pacing maneuvers **(A,C)** and representation of the position of the pacing lead tip in relation to the left Tawara branch for specified types of ventricular capture **(B)**.

The left subclavian approach was preferred per study protocol. The His bundle region was mapped using a SelectSecure™ lead (model 3830, 69 cm, Medtronic Inc., Minneapolis, MN), delivered through a fixed-curve sheath (C315 HIS, Medtronic, Minneapolis, MN), and the His bundle signal was identified. If mapping of the His bundle was not successful, the tricuspid valve annulus was visualized by injection of a contrast agent through the C315 His sheath. The lead was then moved toward the right ventricle, along a line between the HB region or the vertex of the tricuspid annulus and the RV apex. We aimed for RV location where either the “W” morphology was seen in lead V1 or QRS complexes, with a preferably normal heart axis was observed during right septal pacing. Then, the lead was screwed deep into the septum to obtain a position on the left side of the interventricular septum producing nsLBBp during unipolar pacing with outputs up to a maximum of 5 V at 0.5 ms. nsLBBp was confirmed based on a change from nsLBBp-to-sLBBp or nsLBBp-to-LVSP using pacing maneuvers. Three types of ventricular capture were included in the study and are shown in Figure 1 and described as follows:

(1) nsLBBp; i.e., concomitant LBB and myocardial capture was defined by a pseudo-RBBB morphology with the terminal r/R in V1 during pacing with an output of 5 V at 0.5 ms, which changed to sLBBp or LVSP while decreasing the pacing outputs.
(2) sLBBp; i.e., selective capture of the LBB, was observed after decreasing the pacing output from nsLBBp with unchanged V5 R wave peak time (RWPT); however, the QRS complex in V1 changed from qR to rsR or rSR (usually the R during sLBBp was wider than nsLBBp) and the EGM signal became “discrete” (5).
(3) LVSP; i.e., pure myocardial capture of the left septum without LBB capture, that was observed after decreasing the pacing output from nsLBBp, and when after the transition the V5 RWPT was prolonged > 10 ms, usually the R amplitude in V1 also decreased or changed from a terminal r/R morphology during nsLBBp to a terminal rs/Rs morphology (6).

If nsLBBp with a transition to sLBBp or LVSP, was not observed during pacing maneuvers, the implant procedure was marked as the procedure without proved LBB capture.

### UHF-ECG Data Acquisition and Analysis of Other Measured Parameters

**Figure 2:**
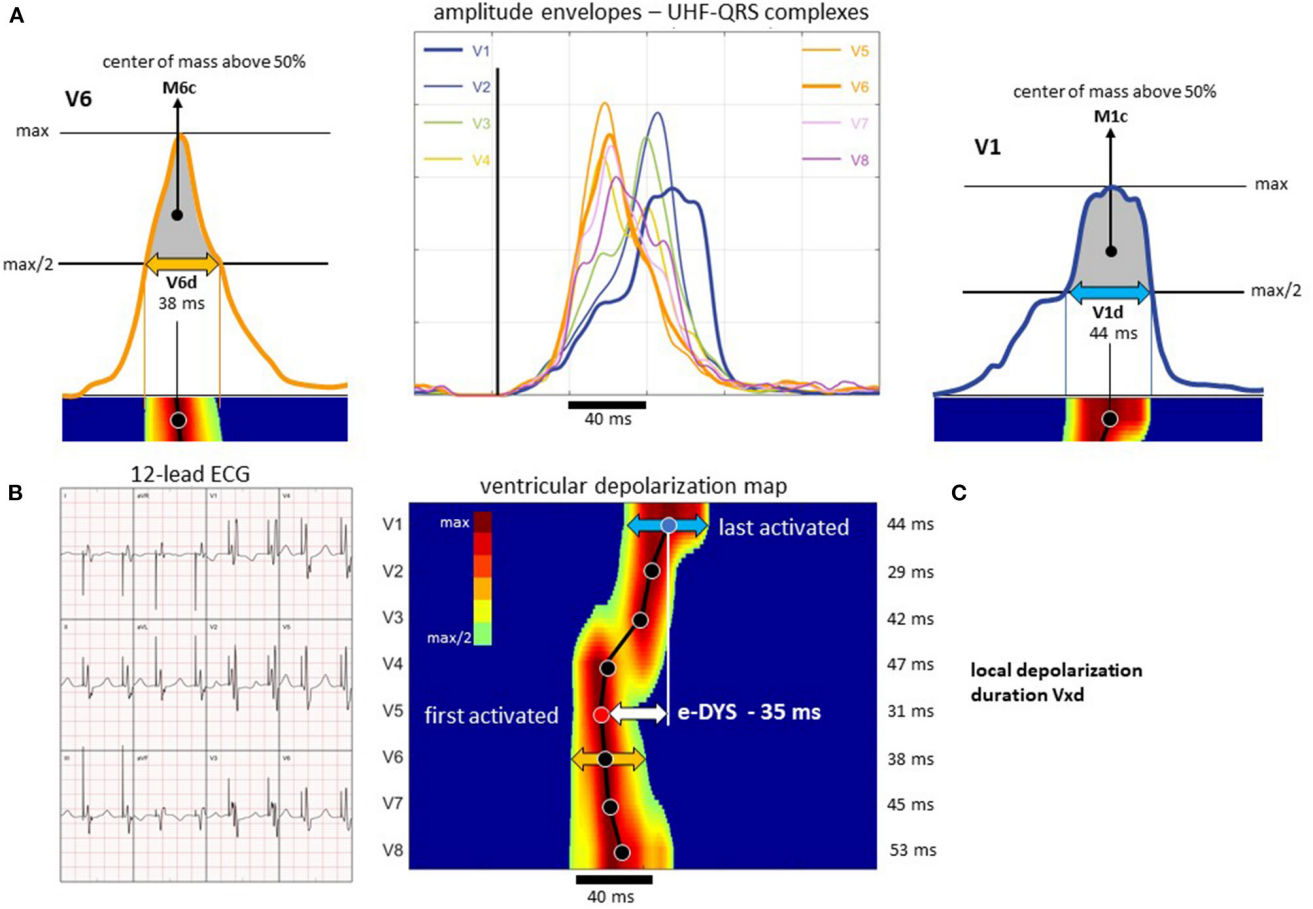
Presentation of the UHF-QRS complexes, Mxc activation times, and Vxd local depolarization duration calculation; patient with sLBBp. **(A)** ultra-high-frequency amplitude envelopes of QRS complexes (UHF-QRS), Mxc computed as the center of mass above 50 percent threshold of the baseline to peak amplitude, Vxd determined as the depolarization duration at the 50 percent threshold of the baseline to peak amplitude. **(B)** 12-lead ECG. **(C)** Ventricular depolarization map with visualization of the M1-8c, electrical interventricular dyssynchrony e-DYS, and the V1-8d values. For details, see Jurak et al. (7). **(C)** The dark line connects the center of masses (solid points) under the specific lead (displayed on the y-axis). Time (ms) is displayed on the x-axis. In this case, the first activation occurred under V5 (M5c), and the last was under V1 (e-DYS = −35 ms). The width of depolarization under V1 is indicated by the blue arrow (V1d), under V6 by the orange arrow (V6d). The numerical parameters of the local depolarization duration (under each lead) are shown on the right side of **(C)**.

A VDI monitor (Ventricular Dyssynchrony Imaging monitor, ISI Brno, Cardion, FNUSA, CZ, 2018) was used to record and analyze the 5 kHz 14-lead ECG signals with a three nV resolution and a frequency range of 1.5 kHz. Standard V1–V8 chest lead positions were used, except for lead V1, which was moved from the fourth to the 5th right parasternal intercostal space to obtain better signals from the lateral RV wall. UHF-ECG data for all captures were collected during 2–3 min of VVI pacing at 110 beats/min. Signal processing and UHF-ECG map construction are described in detail elsewhere (7). Median amplitude envelopes were computed in 16 frequency bands (150–1,000 Hz) for each chest lead. The broad-band QRS complex (UHF-QRS) was constructed as the average of the 16 normalized median amplitude envelopes and displayed as a colored map for V1–V8 leads. The local activation times were calculated as the center of mass (Mxc) of the UHF-QRS above the 50% threshold of the baseline-to-peak amplitude for each chest lead. The local depolarization durations under leads V1–V8 were computed as the UHF-QRS duration at 50% of its amplitude (the Vxd parameter). Interventricular electrical dyssynchrony, i.e., e-DYS (the maximum difference between M1-8c) and Vd_mean_ (mean value of V1-8d), were calculated—Figure 2. A positive e-DYS indicates delayed LV activation, and a negative e-DYS indicates delayed RV activation.

Global QRS durations (QRSd) were measured using an electrophysiology system (Labsystem Pro, Boston Scientific, USA) from the earliest to the last deflection in any of the 12 leads during spontaneous rhythms. During nsLBBp and LVSP, the beginning of the QRS was measured from the pacing artifact (QRSd) and during sLBBp it was measured from the earliest deflection identified after the pacing artifact. The paced V5 RWPT was measured from the pacing artifact to the maximum positive QRS amplitude in lead V5. All measurements were done at 200 mm/s using two consecutive beats, and their average values were taken.

During the procedure, 2–3 ml of contrast agent was injected through a C315 HIS sheath in the LAO projection; lead depth inside the septum was measured using an xViewer (Vidis, Prague, Czech Republic) and the distance between the tip and the anode ring of a 3,830 lead (10.8 mm) in LAO was used as a reference. The QRS axis in the frontal plane was calculated and considered left-deviated if it was −30° to −90°, normal (−29° to 105°), right-deviated (105° to 180°), or extreme deviated (−90° to −180°).

### Statistics

**Figure 3:**
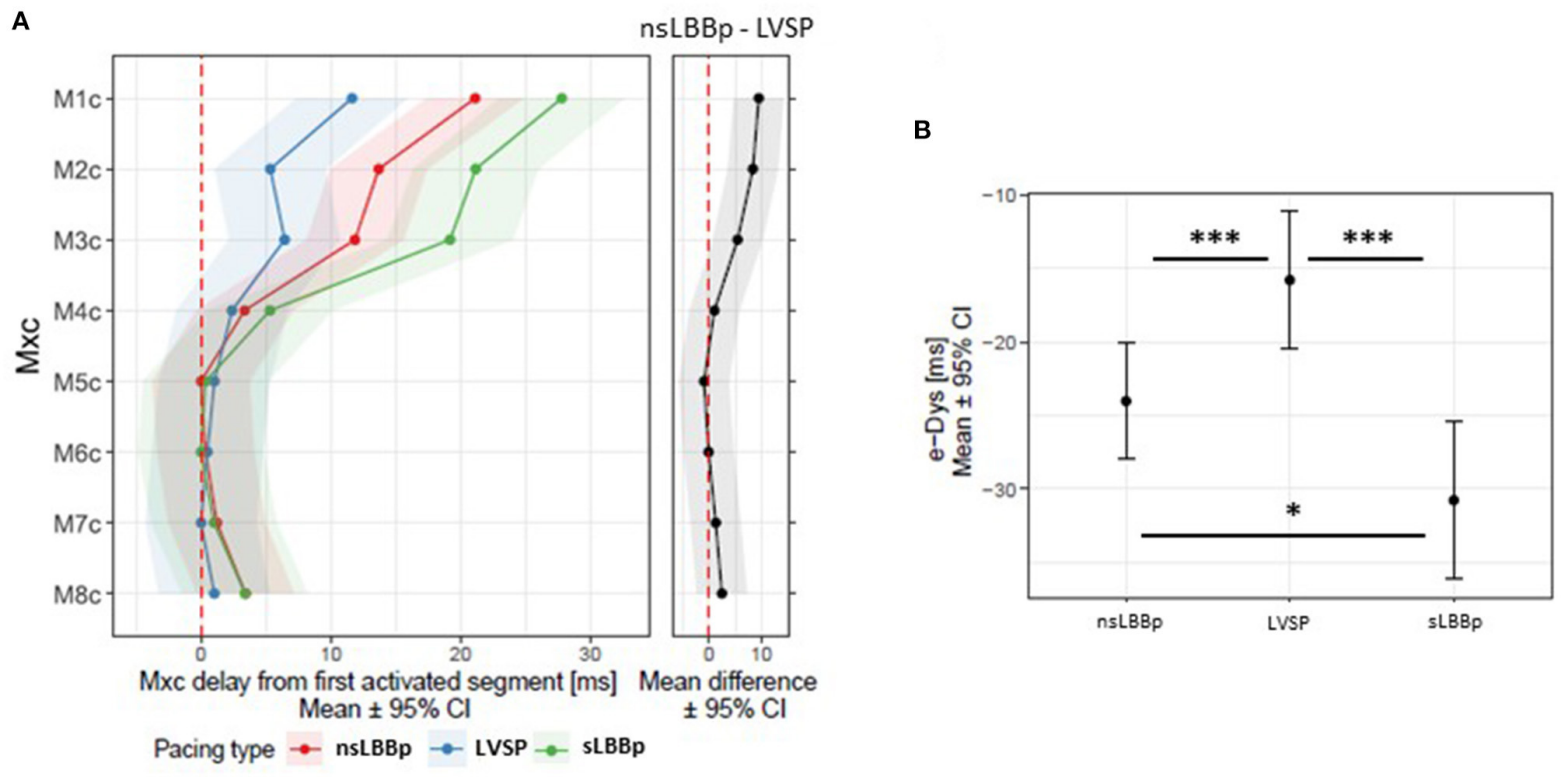
**(A)** Local activation times M1c−8c (first activated segment was placed at 0 ms) and a comparison of e-DYS between nsLBBp, LVSP, and sLBBp **(B)**. *p < 0.05; ***p < 0.001.

**Figure 4:**
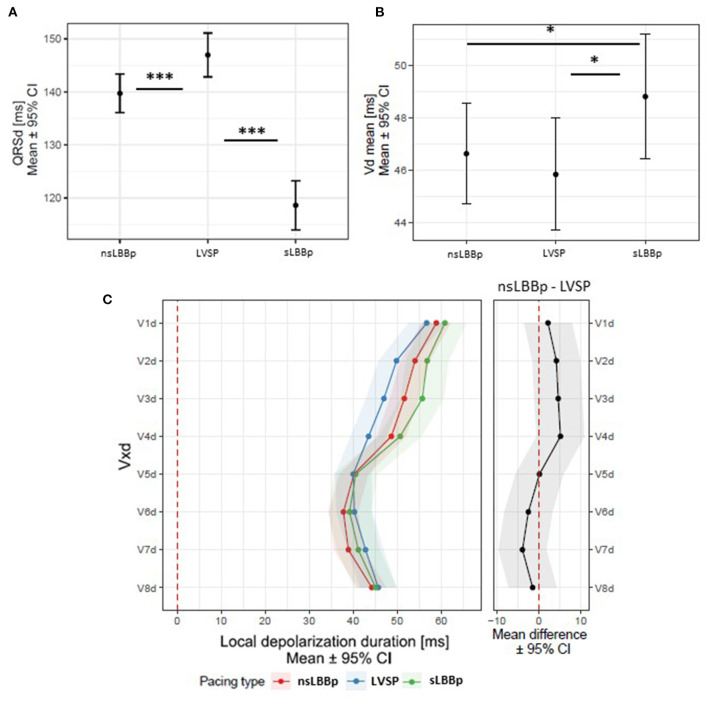
**(A)** QRSd, Vd_mean_ **(B)**, and local depolarization durations (Vd in V1–V8) **(C)** between nsLBBp, LVSP, and sLBBp. *p < 0.05; ***p < 0.001.

**Figure 5:**
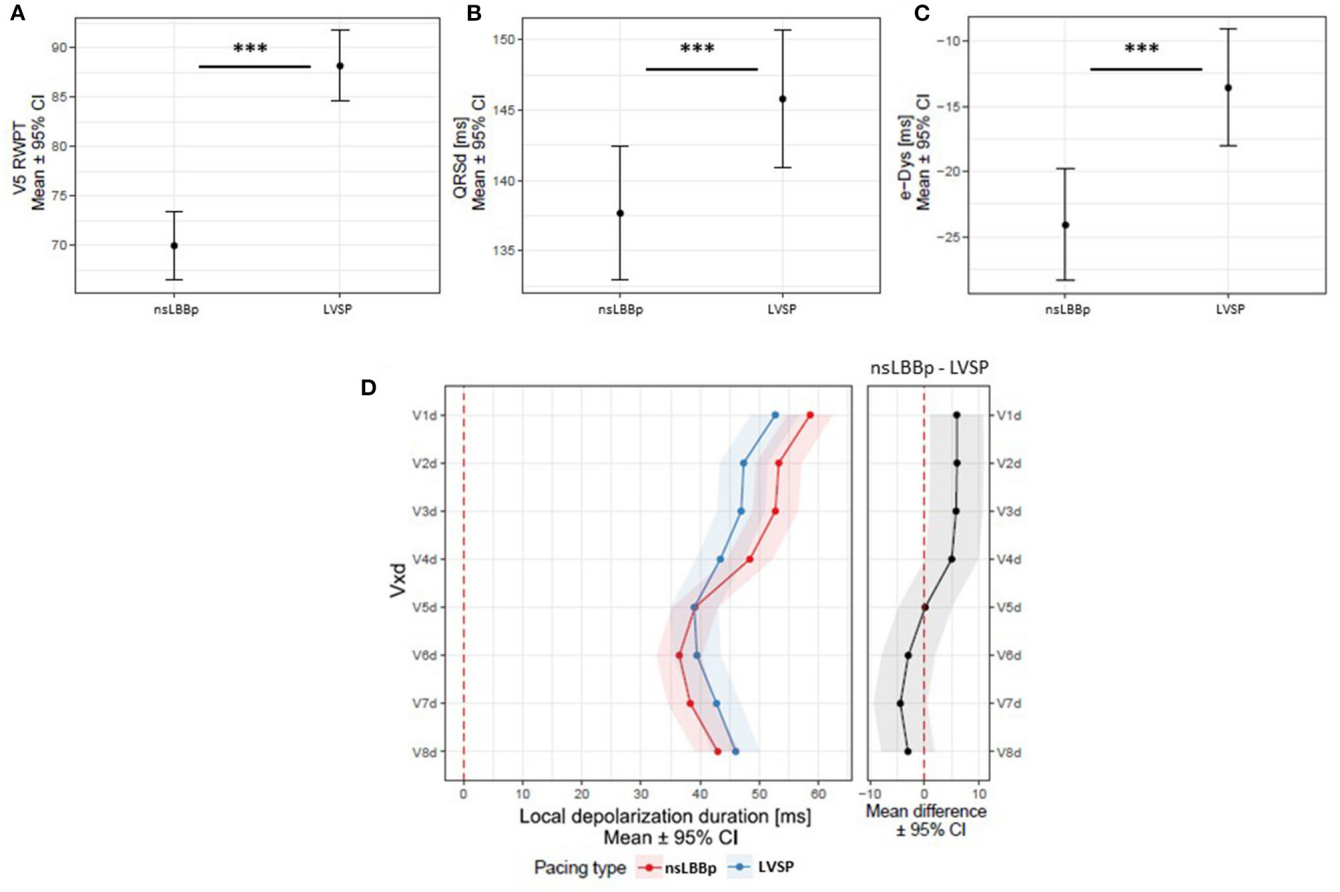
**(A)** V5 RWPT, QRSd **(B)**, e-DYS **(C)**, and local depolarization durations (Vd in V1–V8) **(D)** between nsLBBp and LVSP with normal heart axes. ***p < 0.001.

**Figure 6:**
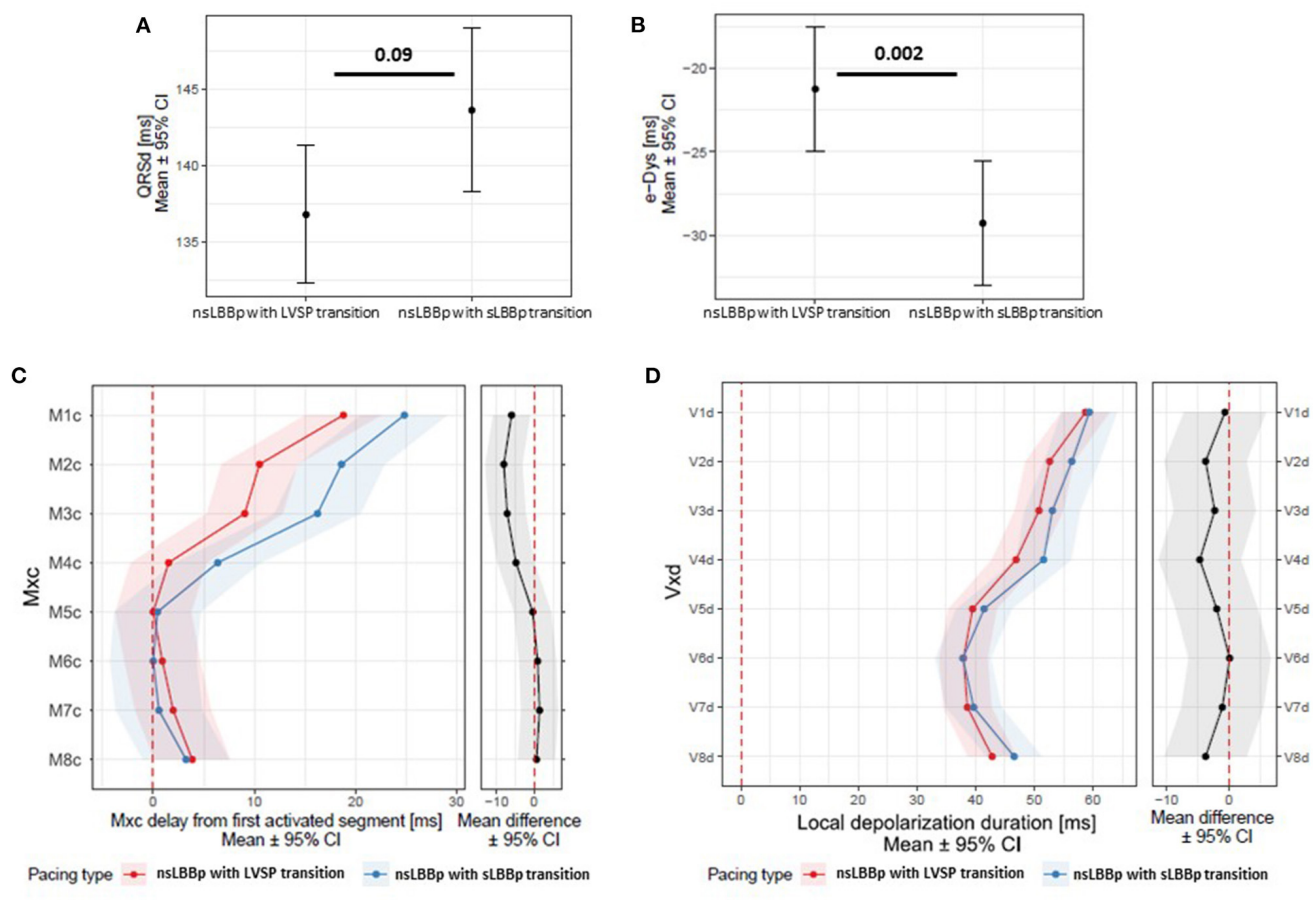
**(A)** QRSd, e-DYS **(B)**, local activation times M1c-8c (first activated segment was placed at 0 ms) **(C)** and in **(D)** local depolarization durations (Vd in V1–V8) between nsLBBp with a transition to LVSP and nsLBBp with a transition to sLBBp while decreasing the pacing output.

An exploratory data analysis was performed for all parameters. Unpaired comparisons of continuous and categorical variables were made using the unpaired *t*-test and Chi-square test. Repeated measurement comparisons were made using a linear mixed effect model (LMEM) and the Tukey multiple comparison test. The results of these models are presented as means with 95% confidence intervals and comparisons as mean differences with 95% confidence intervals and *p*-values (Figures 3–6; Supplementary Figures 1, 2). A *p*-value < 0.05 was considered statistically significant. RStudio version 1.2.1335 with R version 3.6.1 was used to perform statistical analyses. The LMEM was calculated using lme4 version 1.1–21. If not specified, values are shown as means (95% CI).

## Results

Lead placement in the left septal position resulting in nsLBBp that was confirmed using pacing maneuvers was successful in 57 of 96 (59%) patients, and these patients were included in the analyses. Patients without proved LBBp capture were more likely to suffer from heart failure, coronary artery disease, and type 2 diabetes mellitus compared to patients with proved nsLBBp capture (Table 1). Additionally, their septum's were thicker, and the indication for pacing was more often an AV block. In two patients, nsLBBp recordings were inadvertently omitted, and two other patients had two nsLBBp recordings (from different pacing locations). In total, we analyzed 57 nsLBBp, 23 sLBBp, and 34 LVSP in 57 patients with successful nsLBBp implants.

**Table 1:** Clinical characteristics of all patients and patients with successful and unsuccessful nsLBBp implants.

	**All *n* = 96**	**Patients with proved nsLLBp *n* = 57**	**Patients without proved nsLBBp *n* = 39**	***p* value**
Age (years), mean ± SD	77 ± 8	77 ± 8	77 ± 8	0.66
Male gender, *n* (%)	59 (61)	31 (54)	28 (72)	0.09
**Comorbidities**
• Heart failure, *n* (%)	17 (18)	6 (11)	11 (28)	**0.02**
• Coronary heart disease, *n* (%)	37 (39)	16 (28)	21 (54)	**0.01**
• Diabetes mellitus, *n* (%)	41 (43)	18 (32)	23 (59)	**0.007**
• Hypertension, *n* (%)	79 (82)	46 (81)	33 (85)	0.62
LV ejection fraction (%), mean ± SD	56 ± 6	57 ± 6	56 ± 6	0.98
Septal thickness, mean ± SD	11.1 ± 2	10.9 ± 1	11.4 ± 2	**0.03**
**Pacing indications**
• AV block, *n* (%)	57 (59)	28 (49)	29 (74)	**0.01**
• SSSy, *n* (%)	28 (29)	23 (40)	5 (13)	**0.004**
• Bi-, trifascicular block, *n* (%)	8 (8)	4 (7)	5 (13)	0.33
• Atrial fibrillation with planned AV junctional ablation, *n* (%)	3 (3)	2 (4)	0 (0)	ns
**QRS morphology**
• LBBB, *n* (%)	13 (14)	6 (11)	7 (18)	0.29
• RBBB, *n* (%)	23 (24)	11 (19)	12 (31)	0.20
• IVCD, *n* (%)	11 (11)	7 (12)	4 (10)	0.23
• Narrow QRS, *n* (%)	49 (51)	33 (58)	16 (41)	0.10

*SSSy, sick sinus syndrome; AV, atrioventricular; LBBB, left bundle branch block; RBBB, right bundle branch block; IVCD, non-specific intraventricular conduction delay; narrow QRS, QRSd <120 ms. Bold values are used to highlite a significant difference. ns, nonsignificant*.

The LBB potential was not present in 7/23 (26%) patients with nsLBBp-to-sLBBp transition (4 of them with LBBB during spontaneous rhythm), and in 6/34 (18%) patients with nsLBBp-to-LVSP transition (2 with LBBB), *p* = 0.26. Lead tips in patients with LVSP transition were shallower [14.8 mm (13.9; 15.7)] than in patients with sLBBp transition [15.4 (14.5; 16.4], *p* = 0.003). The LV ejection fraction of patients with transition from nsLBBp-to-LVSP was lower [55% (53; 58)] and their septum's tended to be thinner [10.6 mm (10.1; 11.0)] compared to patients with nsLBBp-to-sLBBp transition [59% (58; 61) and 11.2 (10.7; 11.8), *p* = 0.006 and *p* = 0.06 respectively]. The groups did not differ in other clinical characteristics. LVSP had the longest V5 RWPT [86 ms (83; 89)], *p* < 0.001 compared to both nsLBBp [68 ms (65; 71)] and sLBBp [70 ms (67; 73)].

The sequence of ventricular activation under V4–V8 was the same during all three types of ventricular capture. More delayed activation of ventricular segments under V1–V3 led to significant e-DYS prolongation during both nsLBBp and sLBBp (Figure 3). A negative e-DYS, indicating delayed RV depolarization was present in all 23 sLBBp, 57 of 58 nsLBBp, and 29 of 34 LVSP.

sLBBp had the shortest QRSd, but its Vd_mean_ was longer than during both LVSP and nsLBBp. However, local depolarization durations associated with the depolarization of the LV lateral wall (V5–V8d) were the same during all three capture types. Local depolarization durations under V1–V4 were slightly prolonged during sLBBp, although a statistical difference was only reached in V3d for sLBBp vs. LVSP (*p* = 0.01). No differences in V1d−8d were observed between LVSP and nsLBBp (Figure 4).

Similar results with respect to the ventricular depolarization pattern were observed when comparing nsLBBp, LVSP, and sLBBp in patients without LBBB (non-LBBB group) and nsLBBp vs. LVSP in patients with QRSd below 120 ms (narrow QRS group); sLBBp were not included in this analysis because there were only six sLBBp in patients with narrow QRSs (Supplementary Figures 1, 2).

Significant differences in the proportion of patients with a deviated heart axis were observed in studied capture types. Left or extreme axis deviations were the most common during sLBBp (16 of 23 captures (70%), one of them had an extreme axis deviation); a left axis deviation was present in 27 of 58 nsLBBp (47%). For LVSP, most of the paced QRS axes were normal (27 of 34; 79%).

To exclude the possible influence of lead placement in LBB fascicles (which results in heart axis deviation), we compared nsLBBp (*n* = 31) vs. LVSP (*n* = 27) with normal axes. The V5 RWPT and QRSd during LVSP were longer compared to nsLBBp, but both capture types showed the same local depolarization duration in leads V5–V8. However, LVSP resulted in shorter depolarization durations in V1 to V4 (V1d–V4d) than nsLBBp. Moreover, LVSP with a normal axis resulted in less interventricular dyssynchrony than nsLBBp with a normal heart axis (Figure 5C).

As a result of precise lead placement, two different nsLBBp capture types were present. The first with a transition from nsLBBp-to-LVSP, and the second was a transition from nsLBBp-to-sLBBp while decreasing the pacing output. To investigate if they were the same or represented two capture types with different impacts on ventricular depolarization, we compared them to each other. We found no difference in the V5 RWPT [68 ms (65; 71) vs. 69 ms (65; 72), *p* = 0.9], QRSd, or local depolarization duration between them. However, nsLBBp with a transition to LVSP had less delayed activation of myocardial segments under V1–V3 and shorter e-DYS than nsLBBp with a transition to sLBBp (Figure 6).

## Discussion

This study showed that significant differences exist between ventricular captures when pacing the LBB or left septal myocardium in the immediate vicinity of the LBB. They are:

(1) sLBBp and nsLBBp are equivalent with respect to LV depolarization, but sLBBp leads to greater interventricular dyssynchrony than nsLBBp.
(2) Left septal myocardial pacing from the location where nsLBBp could be achieved during increasing the pacing output up to 5 V at 0.5 ms (LVSP) not only preserves interventricular dyssynchrony better than sLBBp and nsLBBp, but it also does not significantly prolong LV lateral wall depolarization times in patients with bradycardia.
(3) Small differences in ventricular activation are present between the two types of nsLBBp based on the transition pattern seen during pacing maneuvers, i.e., nsLBBp with a transition to LVSP leads to less delayed activation in the leads placed above the septum and the right ventricle compared to nsLBBp with a transition to sLBBp.

### Selective and Non-selective Left Bundle Branch Pacing

Pacing of the left bundle branch is a relatively new pacing approach that preserves left ventricular synchrony at the costs of creating left to right interventricular dyssynchrony (8). Two types of LBB pacing have been described. The first one was sLBBp, during which the tissue of the left bundle is exclusively captured. During the nsLBBp, both LBB and adjacent myocardial tissue are captured at the same time. This results in changes in the QRS morphology and EGM signal characteristics (1), and the resultant left ventricular depolarization is a mix of conductive tissue and myocardial activation. As our results showed, there were no differences between these two LBB capture types regarding the sequence of ventricular activation or local depolarization durations under the lead associated with the LV lateral wall depolarization. This suggests that the contribution of myocardial wave-front propagation on LV activation during nsLBBp is minimal, and both capture types should be considered equivalent with respect to LV depolarization. However, the increased delay in RV activation resulted in greater interventricular dyssynchrony during sLBBp. This is very likely the result of concomitant myocardial capture during nsLBBp, which enables earlier trans-septal electrical wave-front propagation and further RV depolarization.

### Left Ventricular Septal Myocardial Pacing

The exact criteria for pacing the left ventricular septum were not established yet. Some studies described the differences in EGM signals, QRS morphology, duration, and paced V6 RWPT between LVSP that emerge from the nsLBBp while decreasing pacing output (1, 9). However, a pseudo-right bundle branch block pattern, usually considered the main marker of left septal pacing, is also present at shallower pacing positions than in location where LVSP transits from nsLBBp (2, 3). As we showed in our previous work on a similar group of patients with bradycardia using the same methodology for lead depth measurement, terminal rs/Rs morphology in V1 during left septal myocardial pacing appeared ~2/3 (10 mm, i.e., 67%) of the distance between the right septum and pacing positions with nsLBBp. Terminal r/R morphology in V1 during left septal myocardial pacing was present on average 4/5 (12 mm, i.e., 81%) of the distance between the right septum and nsLBBp pacing positions (4). These two capture types resulted in less interventricular dyssynchrony but prolonged LV lateral wall depolarization duration compared to nsLBBp. Nonetheless, pacing from these positions did not lead to LBB capture when pacing with outputs up to 5 V at 0.5 ms, and LBBpotential was seen in a minority (7%) of cases. These are the main differences between pacing positions studied previously and left septal pacing with myocardial capture (LVSP) studied in this manuscript. LVSP was observed to be 98% of the distance between the right septum and nsLBBp pacing positions (14.8 mm vs. 15.1 mm), and LBBpotential was observed in a majority of cases (82%). LVSP caused the same interventricular dyssynchrony as left septal myocardial captures with terminal r/R morphology studied previously (4) (on average −16 ms). However, the LV lateral wall depolarization durations using LVSP in close proximity to LBB were shorter and similar to those seen during both sLBBp and nsLBBp. These findings demonstrate differences in ventricular depolarization during various types of left septal myocardial capture. They differ in the degree of interventricular dyssynchrony and the pattern of LV lateral wall activation. The deeper the lead is inserted into the septum during left septal myocardial pacing, the faster the LV lateral wall depolarization is obtained. The main difference in the LV activation pattern seen during LVSP compared to shallower left septal positions with myocardial capture is very likely related to the distance between the pacing lead tip and the left ventricular subendocardial His-Purkinje conductive tissue. With the LVSP in close proximity to LBB, the distance is so small that the contribution of the electrical wave-front originating from activated myocardial cells to LV depolarization is minimal. So, the ventricular depolarization is very similar to that seen during LBBp.

### Non-selective LBBp With LVSP and sLBBp Transition During Pacing Maneuvers

Similar to His bundle pacing (HBp), the lead tip dedicated for LBB pacing can be placed inside the conductive tissue (sLBBp and nsLBBp present during pacing maneuvers) or adjacent to the LBB (LVSP and nsLBBp are seen during pacing maneuvers). In both situations, nsLBB captures are present at higher pacing outputs, and they are not considered different. As we showed, nsLBBp with a transition to LVSP was responsible for smaller interventricular dyssynchrony in our study. This was possibly due to shallow pacing positions with decreased left to right trans-septal conduction times. Compared to HBp, in which the para-Hisian pacing position was well-described and is used in clinical practice (10, 11), reports on LBB pacing suggested preferential lead tip placement in the LBB to obtain sLBBp and nsLBBp at different pacing outputs (12, 13). However, as we have shown in our work, the pacing of the left septum in close proximity to LBB can be an alternative for patients with bradycardia. Both types of captures seen in this location (LVSP and nsLBBp) preserve interventricular synchrony better than captures seen when the lead tip is located inside the LBB (sLBBp and nsLBBp) and does not worsen LV depolarization pattern significantly. It is also worth mentioning that this pacing position is potentially safer for patients due to shallower lead placement, which decreases the risk of lead perforation into the LV.

### Limitations

The results of the study cannot be generalized to patients with heart failure and cardiac resynchronization therapy indication since this study included only patients with an indication for pacemaker implantation due to bradycardia. This study was performed during actual implant procedures. UHF-ECG measurements were taken immediately after the lead was placed in the predefined positions and after confirmation of the type of ventricular capture. We cannot rule out that the resultant damage to conductive and myocardial tissue could have influenced the paced ventricular depolarization patterns. Data were not compared to any other invasive or non-invasive electrocardiographic methods, and no hemodynamic or echocardiographic measurements of mechanical dyssynchrony were performed during the procedure. In the case of three patients with complete persistent AV block of 3rd degree during the procedure, we used the morphology of the escape rhythm to classify them into one of QRS complex morphologies (narrow, LBBB, RBBB, and IVCD). This may have led to incorrect results in some of the analyses presented in the manuscript. In two patients, poor QRS signal quality did not allow for the construction of UHF-ECG maps; therefore, these patients were excluded from the study.

## Data Availability Statement

The raw data supporting the conclusions of this article will be made available by the authors, without undue reservation.

## Ethics Statement

The studies involving human participants were reviewed and approved by Ethics Committee of Faculty Hospital Kralovske Vinohrady, Prague. The patients/participants provided their written informed consent to participate in this study.

## Author Contributions

All authors listed have made a substantial, direct, and intellectual contribution to the work and approved it for publication.

## Funding

This paper was supported by the Charles University Research Program Q38, Research Centre program Nos. UNCE/MED/002 and 260530/SVV/2020, Ministry of Health of the Czech Republic, grant number NU21-02-00584, by the CAS project RVO:68081731, and the European Regional Development Fund - Project ENOCH No. CZ.02.1.01/0.0/0.0/16_019/0000868.

## Conflict of Interest

Some of the participating research institutions have filed a European patent application EP 19212534.2: Method of electrocardiographic signal processing and apparatus for performing the method. The remaining authors declare that the research was conducted in the absence of any commercial or financial relationships that could be construed as a potential conflict of interest.

## Publisher's Note

All claims expressed in this article are solely those of the authors and do not necessarily represent those of their affiliated organizations, or those of the publisher, the editors and the reviewers. Any product that may be evaluated in this article, or claim that may be made by its manufacturer, is not guaranteed or endorsed by the publisher.

## Supplementary Material

Supplementary Figure 1 **(A)** QRSd, e-DYS **(B)**, local activation times M1c−8c (first activated segment was placed at 0 ms) **(C)** and local depolarization durations (Vd in V1–V8) in **(D)** between nsLBBp, LVSP, and sLBBp in patients with non-LBB QRS morphology.

Supplementary Figure 2 **(A)** QRSd, e-DYS **(B)**, local activation times M1c−8c (first activated segment was placed at 0 ms) **(C)** and local depolarization durations (Vd in V1–V8) in **(D)** between nsLBBp and LVSP in patients with narrow QRS morphology (QRSd <120 ms).
