# Supplementary figures and images for: Left Ventricular Myocardial Septal Pacing in Close Proximity to LBB Does Not Prolong the Duration of the Left Ventricular Lateral Wall Depolarization Compared to LBB Pacing
Source: Front Cardiovasc Med. 2021 Dec 7;8:787414. doi: 10.3389/fcvm.2021.787414 (PMC8688808; doi:10.3389/fcvm.2021.787414)

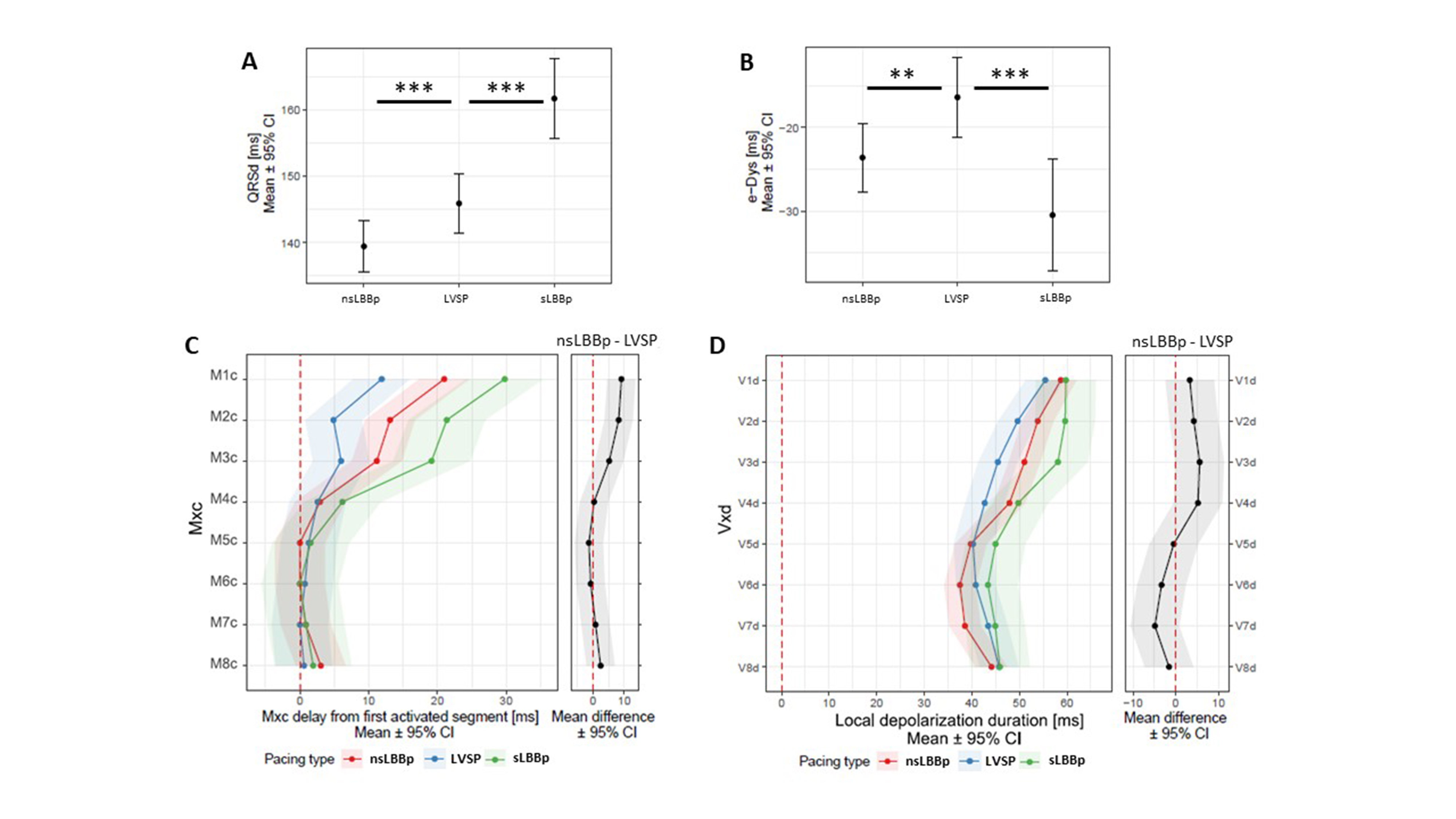

Supplement: Supplementary Figure 1 — (A) QRSd, e-DYS (B), local activation times M1c−8c (first activated segment was placed at 0 ms) (C) and local depolarization durations (Vd in V1–V8) in (D) between nsLBBp, LVSP, and sLBBp in patients with non-LBB QRS morphology. [file Image_1.JPEG]

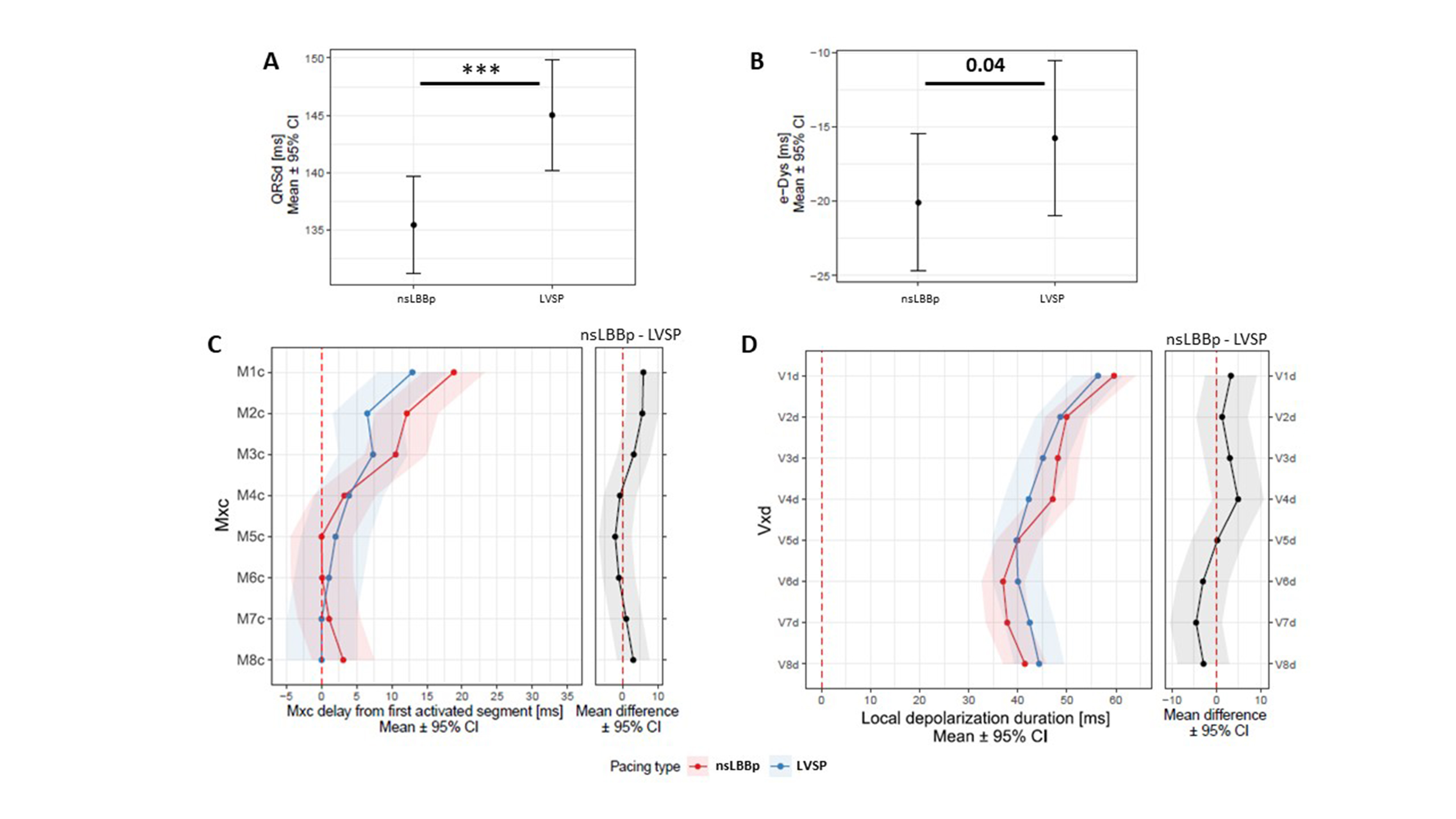

Supplement: Supplementary Figure 2 — (A) QRSd, e-DYS (B), local activation times M1c−8c (first activated segment was placed at 0 ms) (C) and local depolarization durations (Vd in V1–V8) in (D) between nsLBBp and LVSP in patients with narrow QRS morphology (QRSd <120 ms). [file Image_2.JPEG]
